# Supplementary material for: Deep learning of contagion dynamics on complex networks
Source: Nat Commun. 2021 Aug 5;12:4720. doi: 10.1038/s41467-021-24732-2 (PMC8342694; doi:10.1038/s41467-021-24732-2)
Supplement: Supplementary file 1 — Supplementary Information [file 41467_2021_24732_MOESM1_ESM.pdf]

Deep learning of contagion dynamics on complex networks
— Supplementary Information —

Charles Murphy, Edward Laurence, and Antoine Allard

Département de Physique, de Génie Physique, et d'Optique,
Université Laval, Québec (Québec), Canada G1V 0A6 and
Centre interdisciplinaire en modélisation mathématique,
Université Laval, Québec (Québec), Canada G1V 0A6

(Dated: June 23, 2021)

Contents

I. Derivation of the importance weights 2
A. Preliminaries 2
B. Generalizing the importance weights to continuous states 2
1. Neighbor-Dependent Weights 2
2. Reducing the Complexity 3

II. Loss descent patterns 5

III. Impact of hyperparameters 7
A. Performance measures and other metrics 7
B. Time Series Length 7
C. Network size 8
D. Resampling time 10
E. Importance Sampling Bias 10
F. Graph Neural Network Architecture 12
1. Models 12
2. Results 14
3. Graph neural networks on dynamic networks 16

IV. Interpretability of the models 17

Supplementary Note I — Derivation of the importance weights

A. Preliminaries

The importance weight $w_i(t)$ quantifies the extent to which the configuration of a node i at a time t weighs in the loss function. In turn, this affects how the parameters are optimized by correcting further for more important configurations. Yet, it is necessary to correctly define what ”importance” means in this context, otherwise it can lead to badly trained models.

In the main paper, we considered the idea that the configurations should be weighted by an importance sampling (IS) scheme [1] where the target distribution is assumed uniform over all possible configurations. By doing so, we enforce the assumption that all configurations are equally important. Thus, the weights must be inversely proportional to the distribution of these configurations as they are observed in the training dataset. In the context of dynamical processes with a discrete and finite state set $\mathcal{S}$ on simple networks, this observed distribution is simply $\rho(k, x, x_{\mathcal{N}})$, where k is the degree of a node, $x \in \mathcal{S}$ is its state and $x_{\mathcal{N}} \in \mathcal{S}^k$ is the vector state of its neighbors. The importance weight of a node i at time t is then

$$w_i(t) \propto \Big[\rho(k_i, x_i, x_{\mathcal{N}_i})\Big]^{-\lambda} \tag{1}$$

where we allow λ to vary between 0 and 1, corresponding to no IS and pure IS, respectively.

B. Generalizing the importance weights to continuous states

For the metapopulation dynamics, we need to generalize Eq. (1) because the probability distribution ρ in this form can only be evaluated where $\mathcal{S}$ is a finite and countable set: ρ can be computed by counting. When $\mathcal{S}$ is a subset of $\mathbb{R}$, counting cannot really be done directly and efficiently, which in turn prevents us from evaluating ρ. Instead, we must rely on some assumptions in order to evaluate ρ efficiently.

1. *Neighbor-Dependent Weights*

We consider the direct generalization of $\rho(k_i, x_i, x_{\mathcal{N}_i})$ to real numbers. First, we factor $\rho(k_i, x_i, x_{\mathcal{N}_i}) = P(k_i)Q(x_i, x_{\mathcal{N}_i}|k_i)$. By doing so, the dependence of the importance weight with the degree is more conspicuous. Then, we break apart $Q(x_i, x_{\mathcal{N}_i}|k_i)$, because $Q(x, x_{\mathcal{N}}|k)$ must be permutation invariant under the neighbors states. This can be done in various ways, but the simplest one is probably

$$Q(x_i, x_{\mathcal{N}_i}|k_i) = \prod_{j\in\mathcal{N}_i} \left[q(x_i, x_j|k_i)\right]^{1/k_i}, \tag{2}$$

where $q(x, x'|k)$ is the pairwise state probability conditioned on the degree k. Here, the geometric mean ensures that $Q(x, x_{\mathcal{N}}|k)$ does not have an artificially small value for nodes of high degree, as the values of $Q(x, x_{\mathcal{N}}|k)$ should roughly be of the size magnitude, for any degrees. In this context, we interpret $q(x, x'|k)$ as being the probability to observe in the training dataset a node of degree k that is in state x and connected to node in state x'. Therefore, its values must be normalized and bounded by the interval $[0, 1]$.

We make use of kernel density estimators (KDE) [2] with a Gaussian kernel to represent $q(x, x'|k)$. For each value of k, we simply build a different KDE, denoted $\hat{q}(x, x'|k)$. The function $\hat{q}(x, x'|k)$ returns density values, which can have any positive value. Thereby, we normalize it to obtain a probability value such that

$$q(x, x'|k) = \frac{\hat{q}(x, x'|k)}{z_k} \tag{3}$$

where

$$z_k = \sum_{t=1}^{T} \sum_{i\in\mathcal{V}} \sum_{j\in\mathcal{N}_i} I(k_i = k)\hat{q}\big(x_i(t), x_j(t)\big|k_i\big) \tag{4}$$

where $I(\cdot)$ is the indicator function.

Furthermore, the configurations must also be weighted with all additional information, that is with the node and edge attributes, i.e. Φ_i and Ω_{ij}, respectively. This is easily achieved with KDE, where we simply concatenate these attributes to the state pairs, i.e. $q(x, x', \phi, \omega|k)$.

2. *Reducing the Complexity*

For continuous state dynamics such as the metapopulaton one, one would like to compute the importance weights using Eq. (3). Now, the problem with using Eq. (3) is that, for a given KDE function $q(x, x', \phi, \omega|k)$, a lot of samples are used to build it. The evaluation of standard KDE is known to scale like $\mathcal{O}(nm)$, where n is the number of samples used to build the KDE and m is the number of samples on which we wish to evaluate the KDE. Consequently, we need $\mathcal{O}\big[(Nk_{\max}T)^2\big]$ steps in order to evaluate all the normalization constant z_k and all the importance weights. For reasonably lengthy time series with not too large networks, it renders the evaluation of the importance weights very inefficient. This is especially intensive for scale-free networks whose maximum degree is $k_{\max} = \mathcal{O}(N^{\frac{1}{\nu-1}})$ [3], where ν is the exponent of the degree distribution.

To reduce the computational burden of evaluating the importance weights, we consider including additional assumptions. First, we assume that the node and edge attributes are conditionally independent from the state pair. This is equivalent to assuming that the degree encodes all the information needed

to describe the state pairs. This allows us to factor them out of the pairwise state probability such that $q(x, x', \Phi, \Omega|k) = \Sigma(\Phi, \Omega|k)q(x, x'|k)$. We additionally assume that the edge attributes are correctly described by their respective mean taken over the neighbors of the node. This is equivalent to using the strength of the node $\Omega_i = \sum_{j\in\mathcal{N}_i} \Omega_{ij}$. Finally, we assume that, at a given time t, the state of the nodes are also correctly described by the average taken over all the nodes, i.e. $\bar{x}(t) = \frac{1}{N}\sum_{i\in\mathcal{V}} x_i(t)$. Thus, we obtained the form we used in the main paper,

$$w_i(t) = \Big[P(k_i)\Sigma\big(\Phi_i, \Omega_i|k_i\big)\ \Pi\big(\bar{x}(t)\big)\Big]^{-\lambda} \tag{5}$$

where Σ and Π are represented by KDE using a similar strategy to Eq. (3). Those simplifications reduce the complexity of evaluating of the importance weights to $\mathcal{O}(N^2 + T^2)$, a considerable improvement to $\mathcal{O}\big[(Nk_{\max}T)^2\big]$.

Supplementary Note II — Loss descent patterns

In the case of the simple, complex and interacting contagion dynamics, we address a problem similar to a classification problem: For a given input, the model learns to assign it the correct label, i.e. the discrete state to which the node transition to. However, contrary to more standard classification problems, the label that the graph neural network (GNN) model learns to assign is not deterministic. Instead, it is assigned stochastically with a transition probability distribution provided by the dynamical process. This dramatically changes how the cross entropy loss decreases as the training goes on, because it is no longer expected to descend to zero (see Fig. 1(a–c)). What is expected to descend to zero is hopefully the difference between the ground truth transition probabilities and those of the GNN. Hence, choosing an objective function such as the Kullback-Liebler divergence (KLD), denoted $\mathcal{D}$, which is intimately related to the cross entropy loss and that quantifies the difference between two probability distributions, should shed some light as to why the cross entropy loss drops to a non-zero constant value. Consider the KLD between two discrete probability distributions p and q,

$$\mathcal{D}(p||q) = \mathcal{H}(p,q) - \mathcal{H}(p) \tag{6}$$

where $\mathcal{H}(p,q) = -\sum_i p_i \log q_i$ is the cross entropy of p and q, and $\mathcal{H}(p) = -\sum_i p_i \log p_i$ is the entropy of p. It is well known that minimizing $\mathcal{D}(p||q)$ with respect to q yields $\mathcal{D}(p||q) = 0$ when $q = p$, which in turn leads to

$$\min_q \left[\mathcal{D}(p||q)\right] = \min_q \left[\mathcal{H}(p,q) - \mathcal{H}(p)\right] = \min_q \left[\mathcal{H}(p,q)\right] - \mathcal{H}(p) = 0.$$

From this expression, we readily obtain the minimum expected value of the cross entropy loss,

$$\min_q \left[\mathcal{H}(p,q)\right] = \mathcal{H}(p)\,. \tag{7}$$

In a realistic scenario, where the ground truth probabilities are not accessible directly, the entropy $\mathcal{H}(p)$ is also not accessible directly, which prevents the use of the KLD as the objective function altogether. For this reason, we use the cross entropy loss in our experiments involving stochastic dynamics. It is also worth monitoring the average entropy of the GNN outcome, which should, in the event that it is close to the ground truth, be of the same magnitude as the minimum of cross entropy loss.

In Fig. 1, we show an example of loss descent for each stochastic dynamics case. We also show the entropy of the GNN outcome averaged over the training dataset, and show the average Jensen-Shannon distance [4] (JSD), a symmetric version of the KLD, between the ground truth transition probabilities and the GNN predictions.

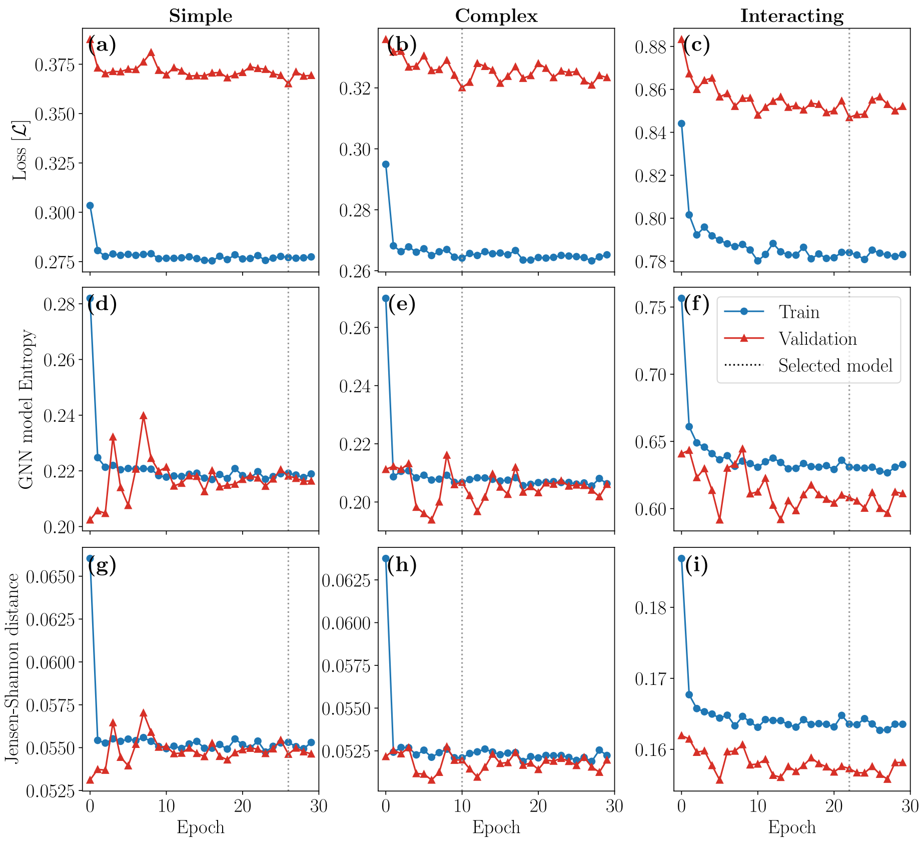

Supplementary Figure 1. **Loss optimization patterns during training.** (a–c) Loss as expressed by Eq. (3) in the main text, (d–f) average entropy of the GNN model predictions, (g–i) average Jensen-Shannon distance (JSD) between the GNN predicted LTPs and the ones given by the MLE. We show the results obtained when using Barabási-Albert networks to generate the data; similar conclusions are obtained when using data generated with Erdős-Rényi networks. All measures shown by these plots are approximated using the importance sampling scheme used to compute the loss. The vertical dotted lines show the minimum value of the validation loss, corresponding to our criterion for the model selection.

Supplementary Note III — Impact of hyperparameters

A. Performance measures and other metrics

In this section, we investigate the impact of several hyperparameters. To quantify the performance of our models, we used two kinds of metrics. The first one is similar to the one we used in the main paper, which is the Pearson error $1 - r$ computed from the Pearson correlation coefficient r between all target-prediction pairs in the dataset. For completeness, the exact definition of r is provided in the Material and Methods section of the main paper. The second one corresponds to the log Jensen-Shannon distance [4] averaged over all target-prediction pairs. The two metrics provide a similar picture of the global performance of the GNN model. Also, because we use discrete state dynamics in this context, we are allowed to evaluate the effective sample size (ESS) in the following way:

$$n_{\text{eff}} = \frac{\left[\sum_{x\in\mathcal{S}}\sum_{\boldsymbol{\ell}} n(x,\boldsymbol{\ell})\right]^2}{\sum_{x\in\mathcal{S}}\sum_{\boldsymbol{\ell}}\left[n(x,\boldsymbol{\ell})\right]^2} \tag{8}$$

where

$$n(x,\boldsymbol{\ell}) = \sum_{i\in\mathcal{V}}\sum_{t=1}^{T} I\Big(x_i(t) = x \wedge \boldsymbol{\ell}_i(t) = \boldsymbol{\ell}\Big) \tag{9}$$

is the number of nodes at any times in the dataset that were in state x and that had a neighborhood state vector $\boldsymbol{\ell}$. To better appreciate the relationship between the performance metrics and the ESS, we center and rescale the ESSs with the mean and standard deviation ESS across the experiments which varies the same hyperparameter.

B. Time Series Length

The time series length, denoted by T, corresponds to the number of time steps in the training dataset. It also affects the length of an epoch. We investigate the values $T = \{100, 500, 1000, 5000, 10000\}$.

Figure 2 shows the accuracy diagrams of GNN models trained using different time series lengths. As we can expect, longer time series tend to yield better models. This is unsurprising for two reasons. First, because the targets with which the models are trained are noisy, it generally helps to have larger a training dataset. Using noisy targets yields a noisy objective function as well, for which the noise can be reduced by increasing the number of samples. Second, using larger datasets means that we train the model for a longer period of time. We also note that, because the gradient descent is performed using a stochastic technique, the results can be a bit inconsistent with our previous observations. This is likely to also affect our next

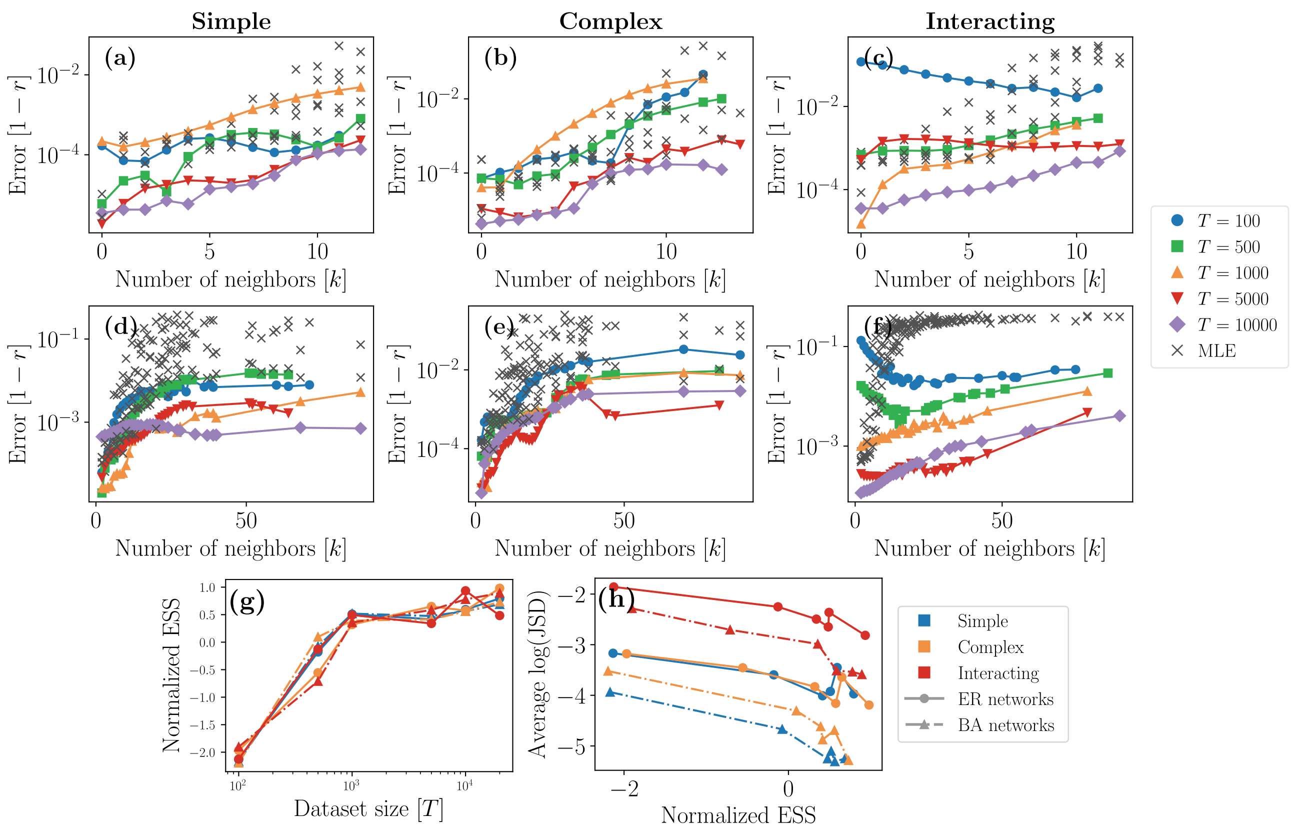

Supplementary Figure 2. Accuracy diagrams for different time series lengths T: We show the accuracy diagrams, that is the error as a function of the degree of the nodes, of GNNs trained on the simple (left column), complex (middle column) and interacting contagion dynamics evolving on Erdős-Rényi (ER, top row) and Barabási-Albert (BA, bottom row) networks. In every panel, we indicate the value of the changing hyperparameter, namely the time series length, with the symbols and the colors according to the legend. The maximum likelihood estimators (MLE), computed from the procedure specified in the main paper, is indicated as a reference. Panel (g) shows the normalized effective sample size (ESS) as a function of the hyperparameter. Finally, panel (h) shows the relationship between the error—the average log-JSD error to be more precise—as a function of the ESS. In panels (g, h), the symbols and line style encode the type of networks used to generate the training dataset and the colors indicate the dynamics.

results, hence we need to keep it in mind. A time-consuming way of addressing this issue would be to train multiple GNNs in the same configurations, and to then average their errors together.

C. Network size

The network size, denoted by N, is the number of nodes in the networks on which the dynamics evolved to generate the training dataset. We investigate the values $N = \{100, 500, 1000, 5000\}$.

Similarly, Fig. 3 shows the accuracy diagrams when changing the network size. At first, increasing

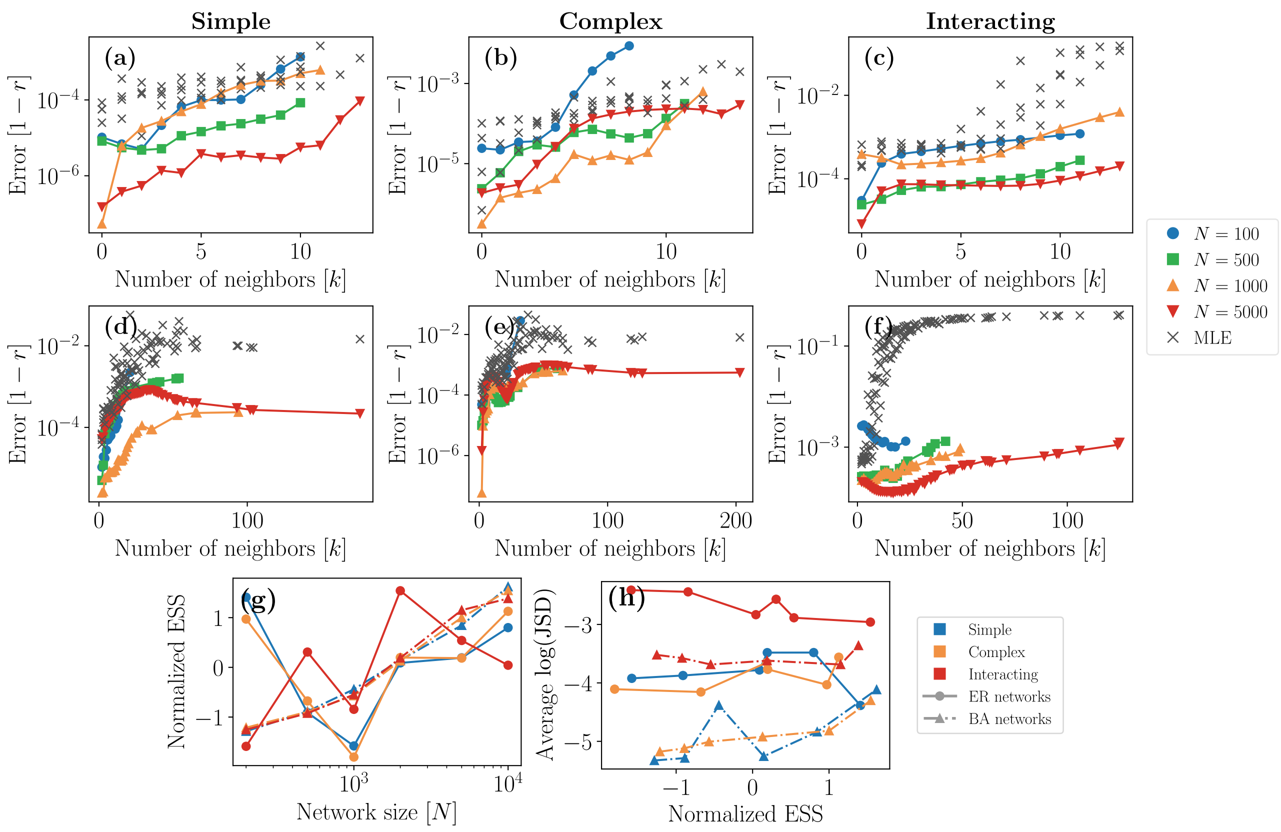

Supplementary Figure 3. **Accuracy diagrams for different network sizes N:** We refer to Fig. 2 for the organization of the panels.

N seems to affect the performance of the models differently depending on the type of networks used. First, for Erdős-Rényi (ER) networks, increasing N does not tend to increase the ESS. This is expected because the maximum degree only slighting increase when the number of nodes is increased, for fixed the average degree $\langle k \rangle$. Hence, we do not observe additional degree classes when N is marginally increased and the training dataset variety remains similar. For Barabási-Albert (BA) networks, we observe something different: While the increase in N leads to higher ESS, there is still no substantial gain in performance. This can be explained by looking at the degree distribution. As more nodes are added to the network, the degree classes get more populated, resulting in increased ESS. However, because the degree distribution is scale-free (with exponent -3), these are not populated evenly and more degree classes are created as N increases.

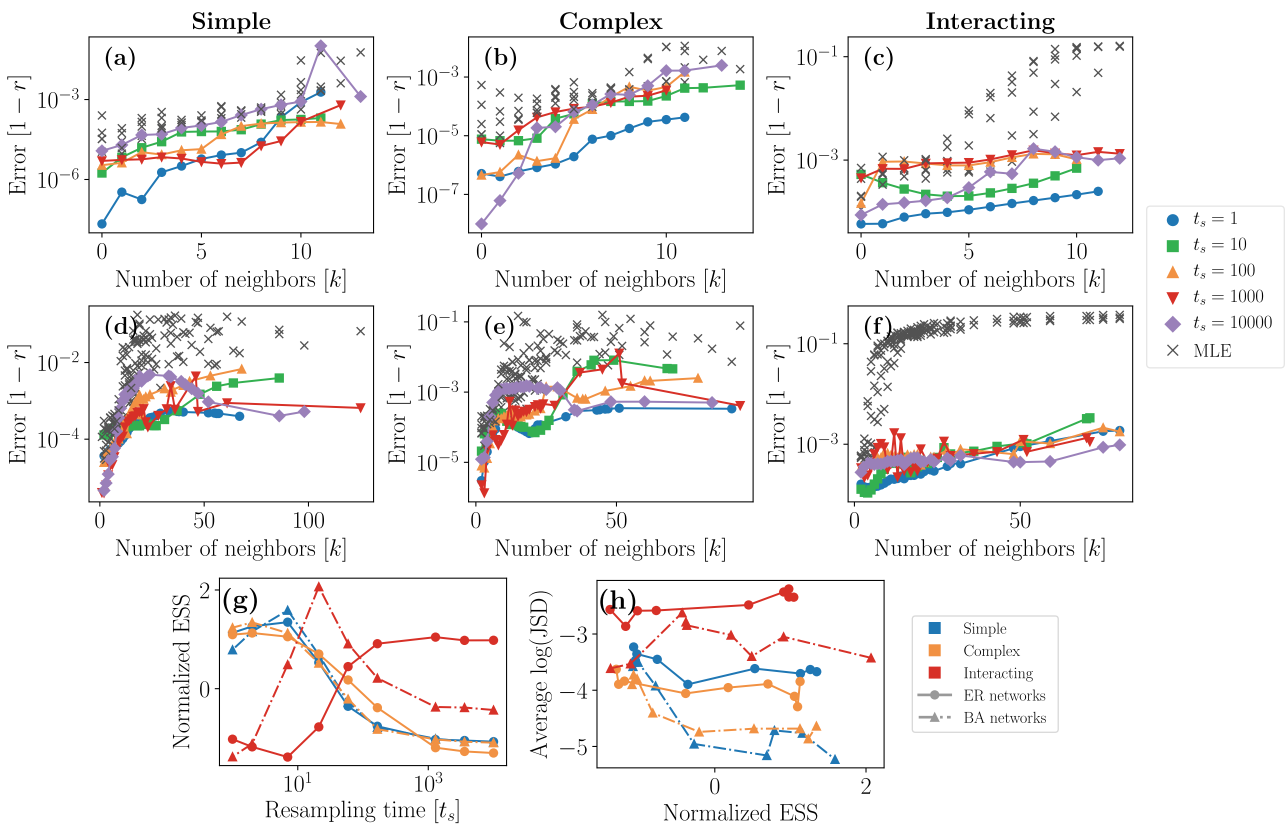

Supplementary Figure 4. **Accuracy diagrams for different resampling times t_s:** We refer to Fig. 2 for the organization of the panels.

D. Resampling time

The resampling time, denoted by t_s, corresponds to the number of time steps before the states of the nodes are reinitialized when generating the training dataset. Recall that this hyperparameter was introduced in the main paper to improve the variability in the dataset, where small values of t_s is expected to increase the ESS. We investigate the values $t_s = \{1, 10, 100, 1000, 10000\}$.

In Fig. 4, we show the accuracy diagrams when the resampling time is changed. It is clear from Fig. 4 that decreasing the resampling time increases the ESS, thus we tend to train better models. However, in most cases, the gain seems to be marginal.

E. Importance Sampling Bias

The role of the importance sampling bias, denoted λ, is to modulate the influence of the importance weights, where $\lambda = 1$ corresponds to the ideal case, which is a standard IS scheme, and $\lambda = 0$ correspond

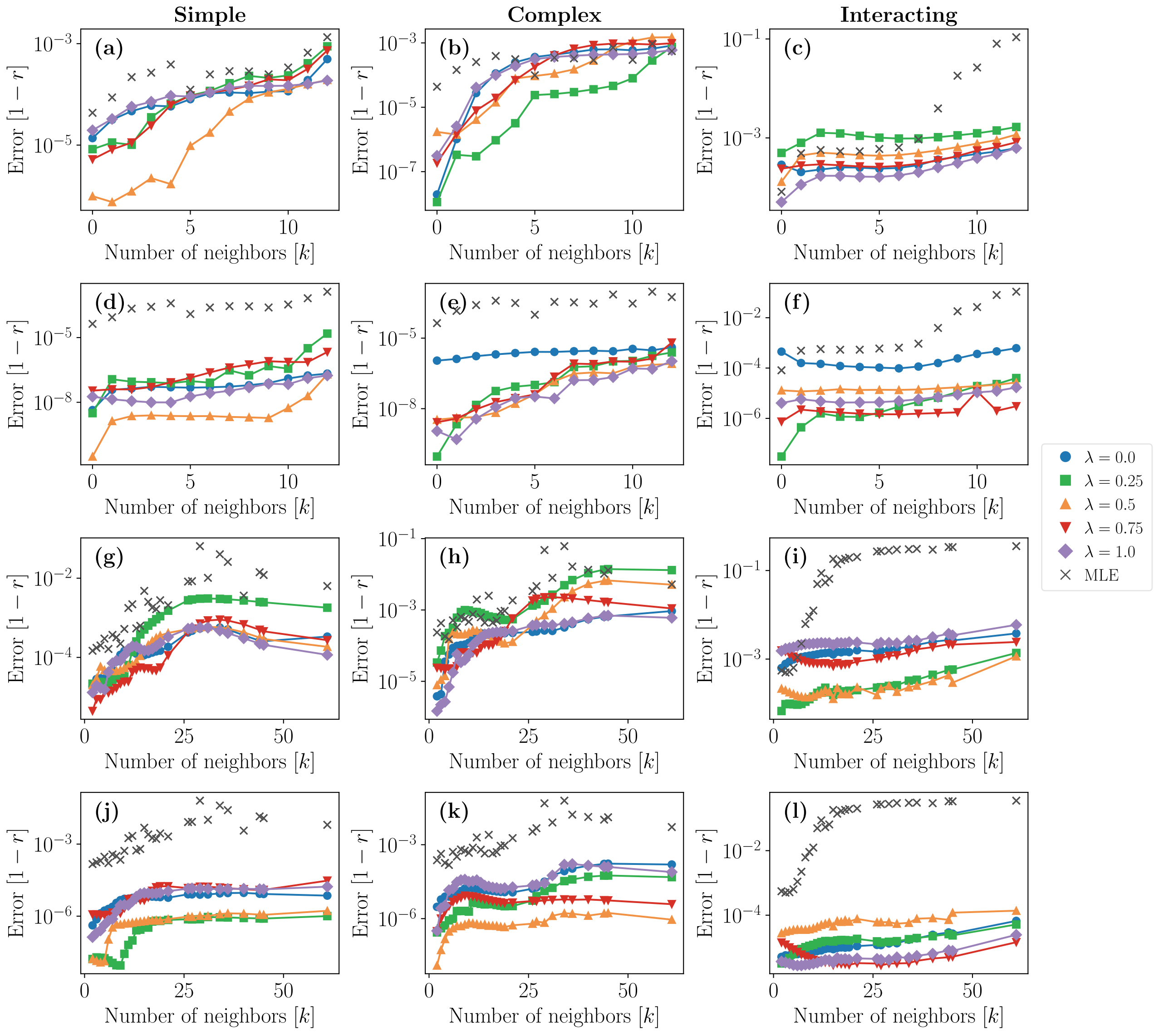

Supplementary Figure 5. Accuracy diagrams for different important sampling bias exponents λ: Similarly to Fig. 2, we show the accuracy diagrams of GNN models trained on (left column) simple, (middle column) complex and (right column) interacting contagion dynamics propagating on (a–f) Erdős-Rényi (ER) and (g–l) Barabási-Albert (BA) networks. We also show the maximum likelihood estimators (MLE) for comparison. Additionally, the panels (a–c) and (g–i) correspond to GNN models trained using the observed outcome, denoted $\tilde{y}_i(t)$ in the main paper, which corresponds to the state of the node at the next time step: the labels are noisy in this case. Conversely, the GNNs corresponding to panels (d–f) and (j–l) used the true transition probabilities, denoted $y_i(t)$ in the main paper: the labels are deterministic in this case. On all panels, the symbols and colors indicate the value of λ as specified by the legend.

to a uniform sampling scheme, that is without IS. There are multiple reasons why it would be preferable to use an exponent $\lambda < 1$. First, it is possible to poorly define the importance weights $w_i(t)$ by a bad choice of assumptions (see Sec. I). This is in part due to the fact that we rely on some statistics to represent the training dataset, which can either contain false assumptions or be poorly estimated due to a small sample size. Second, in the case of stochastic dynamics, letting $\lambda = 1$ is analogous to putting a strong emphasis on rare configurations, which in turn are likely to suffer from a small sample size. This will lead to a poor estimation of the objective function, which is likely to reduce the overall performance of the model. That being said, we investigate the values $\lambda \in \{0, 0.25, 0.5, 0.75, 1\}$.

In Fig. 5, we show the accuracy diagrams when the IS bias exponent λ is changed. To better appreciate the comparison between these different training settings, we used the same dataset, networks and training settings. We also trained our models in two different scenarios: we considered using the observed outcome $\tilde{y}_i(t)$, defined as the state of node i at the next time step, to evaluate the objective function. We also used the true transition probabilities, that is the outcome $y_i(t)$, to evaluate the loss. The difference between these two scenarios is that, in the first case, the targets $\tilde{y}_i(t)$ are noisy, and in the other, the targets $y_i(t)$ are deterministic. As it was mentioned earlier, we argue that the choice of λ will be dependent on the stochastic nature of the underlying dynamics.

First, from Fig. 5, we can see that choosing $\lambda = 1$ rarely leads to the most proficient models, for models trained on both the ER and the BA networks. Only the case of the interacting contagion dynamics does it seem to improve the performance, but as we discussed before, this is conditioned on the fact that either the sample size is large enough or the dynamic is deterministic. Interestingly, the case $\lambda = 0$ often has similar performance as the case $\lambda = 1$ in the noisy scenarios. In general, the best models seem to be obtained when λ is somewhat in the middle, as if the pure IS and the no IS cases are both too strong assumptions.

F. Graph Neural Network Architecture

We investigate the accuracy diagrams of the models when we use different GNN architectures. To be more specific, we consider six additional GNN architectures that has been shown to perform well in the context of structure learning [5–8].

1. *Models*

We label the 6 models as follows: We call our architecture the *Att-GNN*, which is described in detail in the main paper. We also consider the architecture from Ref. [8] with multiple aggregation scheme. This

class of architectures aggregate the neighbors' features as follows:

$$\nu_i = \mathcal{A}(\xi_i) + f_{\mathrm{AGG}}(\{\!\{\mathcal{B}(\xi_j)|j \in \mathcal{N}_i\}\!\}) \tag{10}$$

where $\{\!\{\cdot\}\!\}$ denoted a multiset and we recall that $\mathcal{A}$ and $\mathcal{B}$ are linear transformations with a trainable weight matrix and bias vector. Also, we need to specify the f_{AGG} function, which is a differentiable and permutation-invariant function that aggregates the neighbors' features. We consider three cases for the f_{AGG} function: the mean pooling case, denoted *Mean-GNN*, where

$$f_{\mathrm{AGG}}(\{\!\{x_1, \cdots, x_k\}\!\}) = \sum_{i=1}^{k} \frac{x_i}{k}\,, \tag{11}$$

the max pooling case, denoted *Max-GNN*, where the μth feature is aggregated such that

$$[f_{\mathrm{AGG}}(\{\!\{x_1, \cdots, x_k\}\!\})]_\mu = \max\{x_{\mu,1}, \cdots, x_{\mu,k}\}\,, \tag{12}$$

and the sum pooling case, denoted *Sum-GNN*, where similar to the mean pooling case,

$$f_{\mathrm{AGG}}(\{\!\{x_1, \cdots, x_k\}\!\}) = \sum_{i=1}^{k} x_i\,, \tag{13}$$

Then, we consider four additional standard architectures: the *GraphSage* architecture from Ref. [9], the graph convolution network (denoted *GCN*) from Ref. [5], the original graph attention network (denoted *GAT*) from Ref. [7] and a GNN architecture used to forecast COVID-19 [10] (denoted *Kapoor-GNN*). The GraphSage aggregates the neighbors' features similarly to the Mean-GNN:

$$\nu_i = \boldsymbol{W}_1 \xi_i + \boldsymbol{W}_2 \sum_{j \in \mathcal{N}_i} \xi_j\,, \tag{14}$$

which, in turn is similar to the GCN,

$$\nu_i = \boldsymbol{W} \sum_{j \in \mathcal{N}_i \vee \{i\}} \frac{\xi_j}{(k_i+1)(k_j+1)}\,. \tag{15}$$

Here, $\boldsymbol{W}$ and $\boldsymbol{W}_i$ are a trainable weight matrix. The GAT architecture aggregates the neighbors' features as follows:

$$\nu_i = \boldsymbol{W} \sum_{j \in \mathcal{N}_i \vee \{i\}} a_{ij} \xi_j \tag{16}$$

where

$$a_{ij} = \frac{e^{\theta_{ij}}}{\sum_{j \in \mathcal{N}_i \vee \{i\}} e^{\theta_{ij}}} \tag{17}$$

and

$$\theta_{ij} = \mathrm{LeakyReLU}_{\alpha}\Big(\boldsymbol{a}^T \boldsymbol{W} \xi_i + \boldsymbol{b}^T \boldsymbol{W} \xi_j\Big) . \tag{18}$$

In this case, $\boldsymbol{a}$ and $\boldsymbol{b}$ are weight vectors and $\mathrm{LeakyReLU}_{\alpha}$ is an activation function such that

$$\mathrm{LeakyReLU}_{\alpha}(x) = \begin{cases} x & \text{if } x > 0 \\ \alpha x & \text{otherwise} \end{cases} , \tag{19}$$

and α, the negative slope, is generally fixed to 0.2. Finally, we consider the Kapoor-GNN architecture which was used to forecast COVID-19 in this US at the county level based on the mobility flow [10] between counties. This architecture is composed of a sequence of two GCN layers in series. As the first layer aggregates the features of the first neighbor, the second adds that of the second neighbors as well.

2. *Results*

In Fig. 6, we show the predicted transition probabilities for the simple and complex contagion dynamics. We see that, in general, the standard GNN architectures yield poor performance in predicting the infection probabilities, even though they have been trained using the same dataset, networks and hyperparameters. We believe this is due to the fact that they internally use a non-extensive aggregation operator—for instance mean-pooling and max-pooling, whose output does not scale with the size of the input. To be clearer, let us assume a node of degree k of which we wish to aggregate the features of its k neighbors. By using a non-extensive aggregator, the output is expected to be of a similar scale as that of any other node of degree k'. Hence, the GNN model is likely to have a hard time distinguishing the vector features of nodes of different degrees—a structural feature that we know has a huge impact on most of the dynamical processes on networks. This is in part why almost all GNN architectures described above fail at learning and representing contagion dynamics. The only ones that perform similarly are the Att-GNN and Sum-GNN, which both use extensive aggregators.

From Figs. 6 and 7, we can also appreciate how some GNN architectures are better than others at predicting the recovery probability, this is independent from the neighbors' states unlike the infection probability. Specifically, the GraphSage and GCN architectures have a hard time predicting the recovery probabilities. They may be due to the fact that their aggregator does not distinguish the different values of neighbor features and accept all contributions equally, as opposed to for instance the GAT which is expected to weigh the neighbors' features before aggregating them. The same principle applies to the other architectures that predict correctly the recovery probability. Also, while the Kapoor-GNN, which we recall is composed of

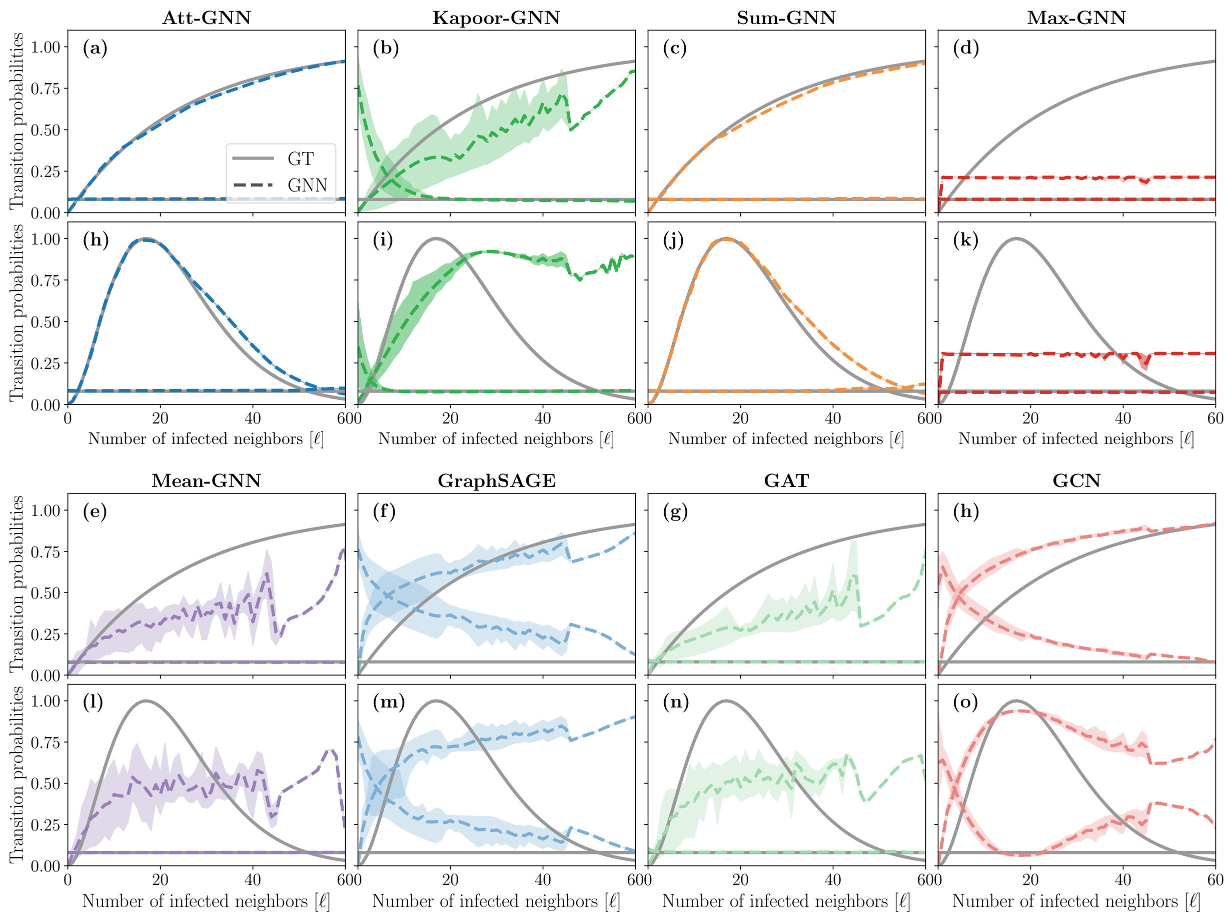

Supplementary Figure 6. **Prediction of different GNN architectures on (a–h) simple and (i–o) complex contagion dynamics on Barabási-Albert Networks:** We show the infection and recovery probabilities as predicted by the trained GNNs (dashed lines), and given by the ground truth (GT, solid lines). Each column corresponds to a different architecture. In the top and bottom rows, all models have been trained on the same training dataset and networks. The training settings and parameters of the dynamics are the same as described in the main paper. Also, we used the same training dataset and networks to train each GNN architecture.

two GCN layers in series, seem to perform better than the single GCN architecture, it still struggles overall in comparison with the Att-GNN. Hence, it suggests that increasing the depth of the GNN aggregation scheme may be insufficient to improve the accuracy of the models.

In summary, not all GNN architectures are capable of learning a dynamical process on networks, which also supports the idea, presented in Ref. [11], that most GNN architectures in fact do not have a high expressive power. Then, we can ask if having an extensive aggregator will always be sufficient in the context of dynamical process learning. From our work, it seems to be the case, but the few examples we provide in this paper are far from conclusive in that regard. However, in the case where extensive aggregator would be insufficient, one could consider new strategies such that which is presented in Ref. [12], where

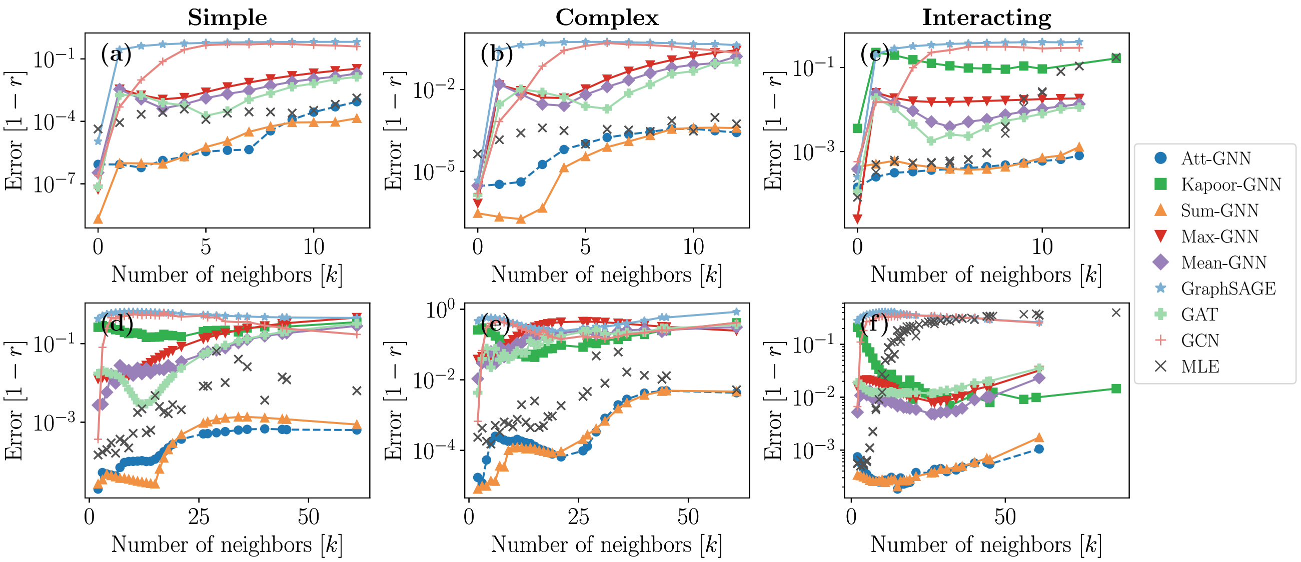

Supplementary Figure 7. **Accuracy diagrams for different GNN architectures:** On each panel, the different GNN architectures were trained on the same dataset with the same training settings and hyperparameters. For further details, we refer to Fig. 2.

multiple aggregators are used in parallel.

3. *Graph neural networks on dynamic networks*

It is worth mentioning that there exists a wide variety of GNN architectures designed specifically to handle dynamic networks [13], i.e. networks whose topology evolves over time. These architectures are typically composed of a sequence of GNN layers each of which being applied to one element of a sequence of temporally ordered networks $(\cdots, G_{t-1}, G_t, G_{t+1}, \cdots)$. These learned representations are then combined to obtain some network and/or node embeddings containing temporal information. While useful for temporal networks, this class of models could hardly be adapted to the task of dynamics learning on *static* networks where it is solely the states of nodes that evolve over time—such as in Figs. 6 and 7. They could, nevertheless, be useful in the context of adaptive systems, where the topology of the network changes over time according to the dynamics of the nodes [14]. However, we suspect that the GNN layers at their core should also be chosen carefully to avoid the complications caused by standard GNN architectures in the context of dynamics learning.

Supplementary Note IV — Interpretability of the models

Like most deep learning models, ours suffer from an interpretability problem in that the parameters learned during training can hardly be associated with specific mechanisms guiding the dynamics. This is in contrast with mechanistic models where the parameters are chosen beforehand to emulate a specific, interpretable behavior. The interpretability problem of our models surely is a drawback of the method, but it can be slightly alleviated. First, as we have demonstrated in the main paper (see Fig. 3), our models can be used to recover the phase transition bifurcation diagrams. As a result, even though the learned parameters of the model are non-interpretable, the behavior of the model, and more specifically the influence of the structure on the dynamics, can still be investigated.

Second, we argue in the Material and Methods section of the main paper that the parameters of our attention mechanism are partially interpretable [15, 16]. Recall that the attention mechanism computes attention coefficients a_{ij} that weigh the interaction between some node features ξ_i and that of one of its neighbors, ξ_j. Hence, we should expect a_{ij} to be high—close to 1—when two nodes are expected to interact, and it should be low—close to 0—otherwise. For example, in simple contagion dynamics, two nodes interact strictly when the target node is susceptible and the source node is infected. Otherwise, the transition probabilities of a target node v_i are invariant with respect to the states of its neighbors, $x_{\mathcal{N}_i}$. We say that the state of a node is neighbor invariant when the transition probability of this node is independent of the state of its neighbors.

In reality, it is not exactly what happens, as we can see in Fig. 8. In fact, when the target nodes are in a neighbor-invariant state (I for the simple and complex contagion dynamics, and I_1I_2 for the interacting contagion dynamics), the attention coefficients are correctly close to zero. However, when they are not (e.g. state S for the simple and complex contagion dynamics), the attention mechanism can be non-zero regardless of the state of the neighbors. We think this is due to the possibility that the representations combined by the attention mechanism are not sparse: the features of the neighbors of a node are combined in such a way that they cancel out the contributions of the features of the noncontributing neighbors. This way, the attention coefficients are not necessarily constrained to be zero even though the nodes are effectively not interacting together. This degeneracy seems to be amplified with a greater number of parallel attention modules, i.e. the number of different available representations learned by the model. This phenomenon is analogous to the sparsity problem in under-determined linear regression models, where multiple parameters can fit the same training dataset depending on the loss function [17]. The addition to the loss function of a L1-norm regularization on the attention coefficients is therefore a promising avenue to increase the

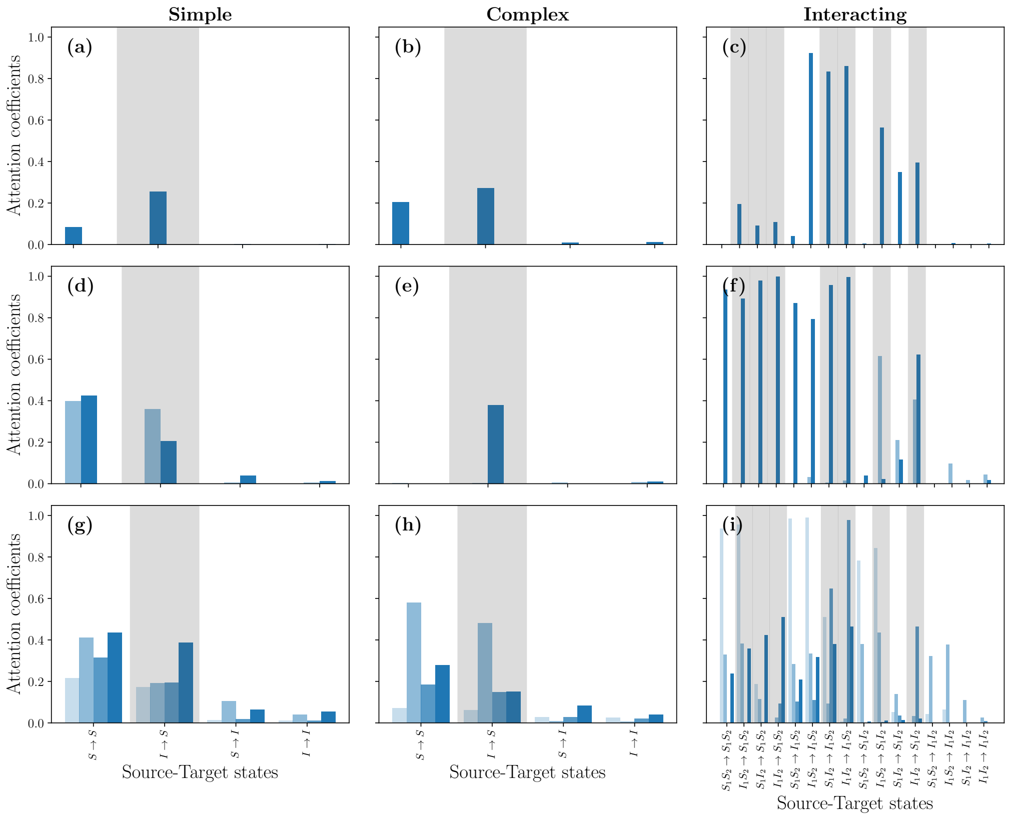

Supplementary Figure 8. **Attention coefficients as a function of the source-target states for (a-d-g) simple contagion, (b-e-h) complex contagion and (c-f-i) interacting contagion dynamics**. We show the attention coefficients of different models: (a–c) are models with one attention layer, (d–f) have two attention layers and (g–i) have four. The values of the different attention layers are shown by the increasingly lighter colored bars. For the source-target states, we indicate the type of node using the directionality of the arrows: for $X \to Y$, X is state of the source node and Y is the state of the target node. We also highlight the source-target states where we expect the transition probability of the target node to be non neighbor invariant. The other hyperparameters are given in Tab. 1 of the main paper and in Sec. A6.

interpretability of our approach.

Supplementary References

[1] R. Y. Rubinstein and D. P. Kroese, *Simulation and the Monte Carlo Method*, 3rd ed. (Wiley, 2016) p. 414.

[2] W. J. Conover, *Practical nonparametric statistics* (John Wiley & Sons, 1998) p. 350.

[3] M. Boguñá, R. Pastor-Satorras, and A. Vespignani, "Cut-offs and finite size effects in scale-free networks," Eur. Phys. J. B **38**, 205–209 (2004).

[4] T. M. Cover and J. A. Thomas, *Elements of Information Theory*, 2nd ed. (Wiley-Interscience, 2005) p. 776.

[5] T. N. Kipf and M. Welling, "Semi-Supervised Classification with Graph Convolutional Networks," (2016), arXiv:1609.02907.

[6] W. L. Hamilton, R. Ying, and J. Leskovec, "Representation Learning on Graphs: Methods and Applications," (2017), arXiv:1709.05584.

[7] P. Veličković, G. Cucurull, A. Casanova, A. Romero, P. Liò, and Y. Bengio, "Graph Attention Networks," (2018), arXiv:1710.10903.

[8] C. Morris, M. Ritzert, M. Fey, W. L. Hamilton, J. E. Lenssen, G. Rattan, and M. Grohe, "Weisfeiler and Leman Go Neural: Higher-order Graph Neural Networks," (2018), arXiv:1810.02244.

[9] W. L. Hamilton, R. Ying, and J. Leskovec, "Inductive representation learning on large graphs," in *Proceedings of the 31st International Conference on Neural Information Processing Systems*, NIPS'17 (2017) p. 1025–1035.

[10] A. Kapoor, X. Ben, L. Liu, B. Perozzi, M. Barnes, M. Blais, and S. O'Banion, "Examining covid-19 forecasting using spatio-temporal graph neural networks," (2020), arXiv:2007.03113.

[11] K. Xu, W. Hu, J. Leskovec, and S. Jegelka, "How Powerful are Graph Neural Networks?" (2018), arXiv:1810.00826.

[12] G. Corso, L. Cavalleri, D. Beaini, P. Liò, and P. Veličković, "Principal Neighbourhood Aggregation for Graph Nets," (2020), arXiv:2004.05718.

[13] J. Skarding, B. Gabrys, and Musial K., "Foundations and modelling of dynamic networks using dynamic graph neural networks: A survey," (2020), arXiv:2005.07496.

[14] Thilo Gross and Bernd Blasius, "Adaptive coevolutionary networks: a review," J. R. Soc. Interface **5**, 259–271 (2008).

[15] S. Vashishth, S. Upadhyay, G. S. Tomar, and M. Faruqui, "Attention interpretability across nlp tasks," (2019), arXiv:1909.11218.

[16] S. Serrano and N. A. Smith, "Is attention interpretable?" in *Proceedings of the 57th Annual Meeting of the Association for Computational Linguistics* (Association for Computational Linguistics, Florence, Italy, 2019) pp. 2931–2951.

[17] R. Tibshirani, "Regression shrinkage and selection via the lasso," J. R. Stat. Soc. B **58**, 267–288 (1996).
